# Supplementary material for: Characterization of Fluidic-Barrier-Based Particle Generation in Centrifugal Microfluidics
Source: Micromachines (Basel). 2022 May 31;13(6):881. doi: 10.3390/mi13060881 (PMC9228483; doi:10.3390/mi13060881)
Supplement: Supplementary file 1 [file micromachines-13-00881-s001.zip › micromachines-1738382-supplementary.pdf]

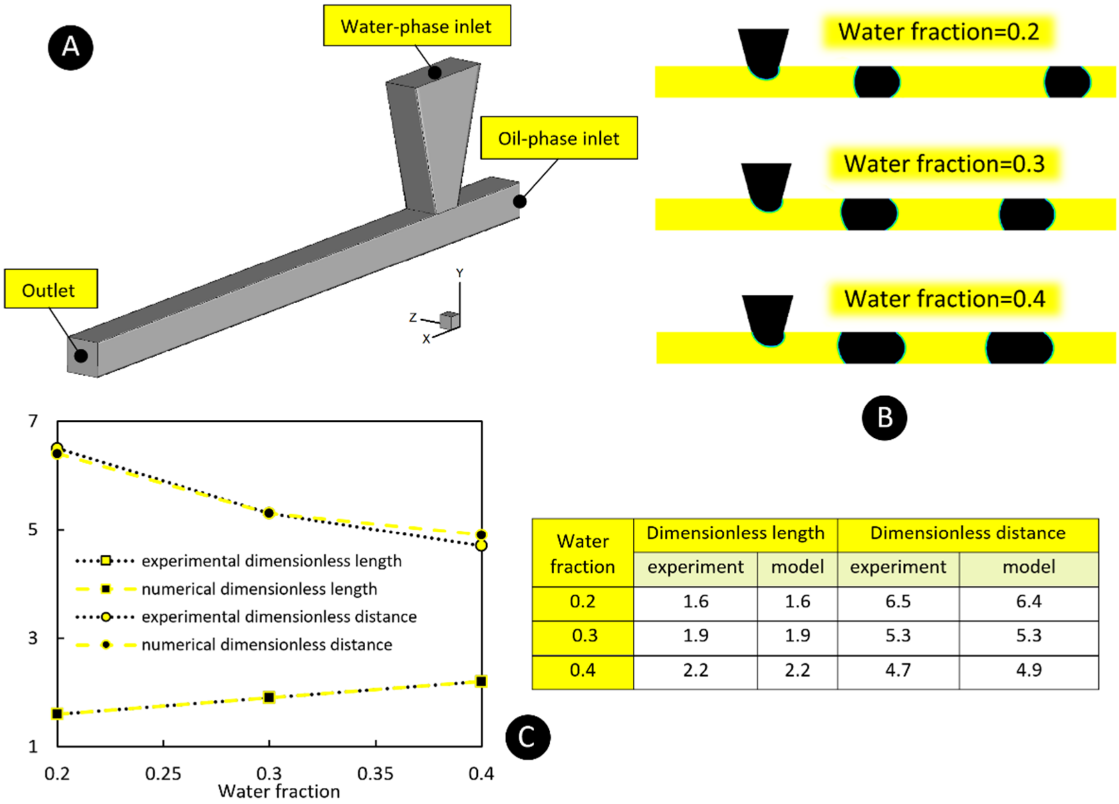

Water fraction	Dimensionless length		Dimensionless distance	
	experiment	model	experiment	model
0.2	1.6	1.6	6.5	6.4
0.3	1.9	1.9	5.3	5.3
0.4	2.2	2.2	4.7	4.9

Figure S1. The results of the multiphase model are verified with the benchmark experimental work done by Tice et al. [38]. As represented in (**A**), water-as the dispersed phase-goes into a microchannel loaded with oil and makes microdroplets. Three different water fractions (the ratio of water flow rate to the total flow rate) of 0.2, 0.3, and 0.4 are considered. The dimensionless distance between the droplets and droplets' dimensionless length are determined based on a channel depth of 40 microns. As displayed in (**B**,**C**), all results are in excellent agreement with the experimental data.
